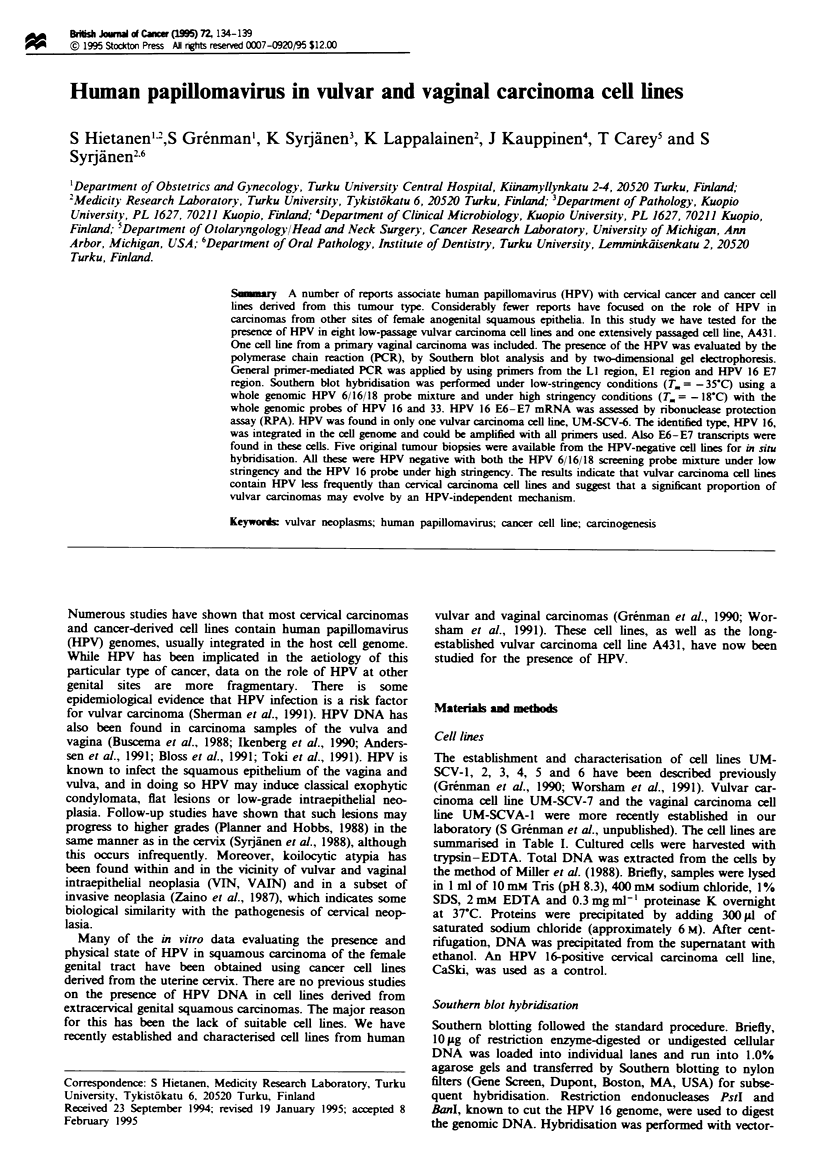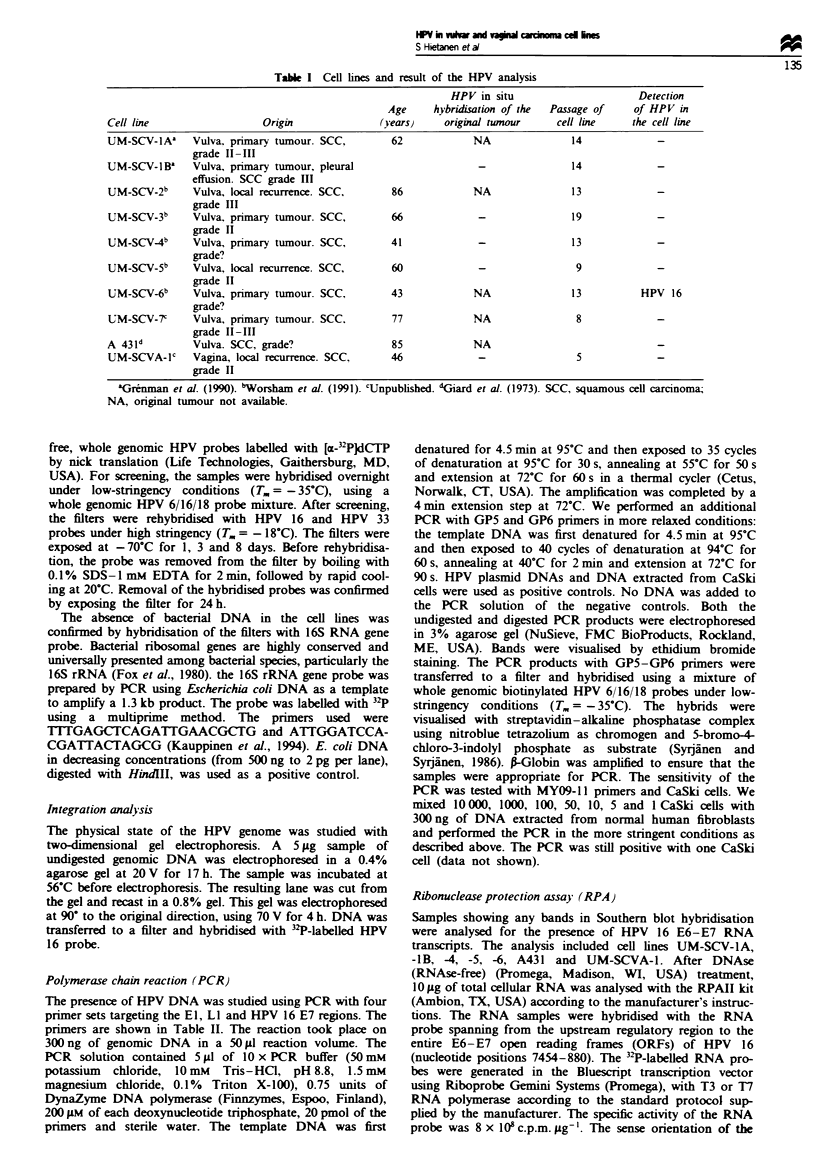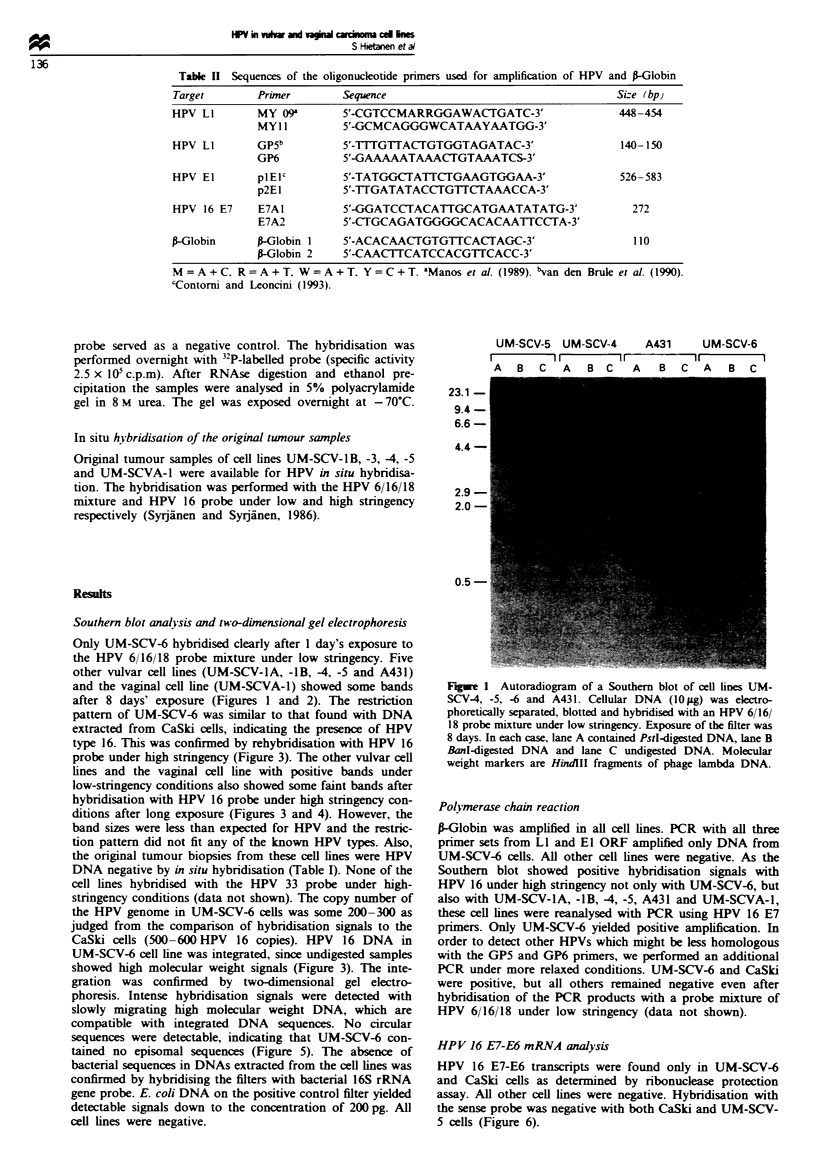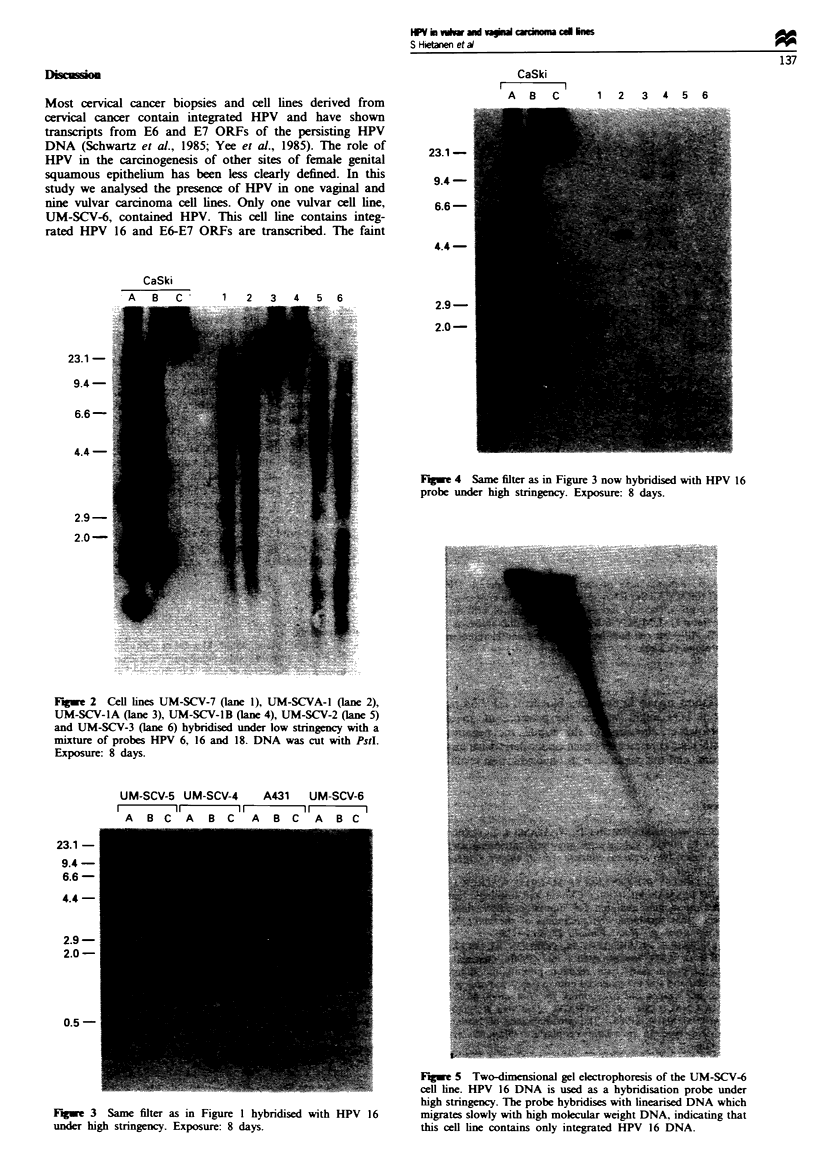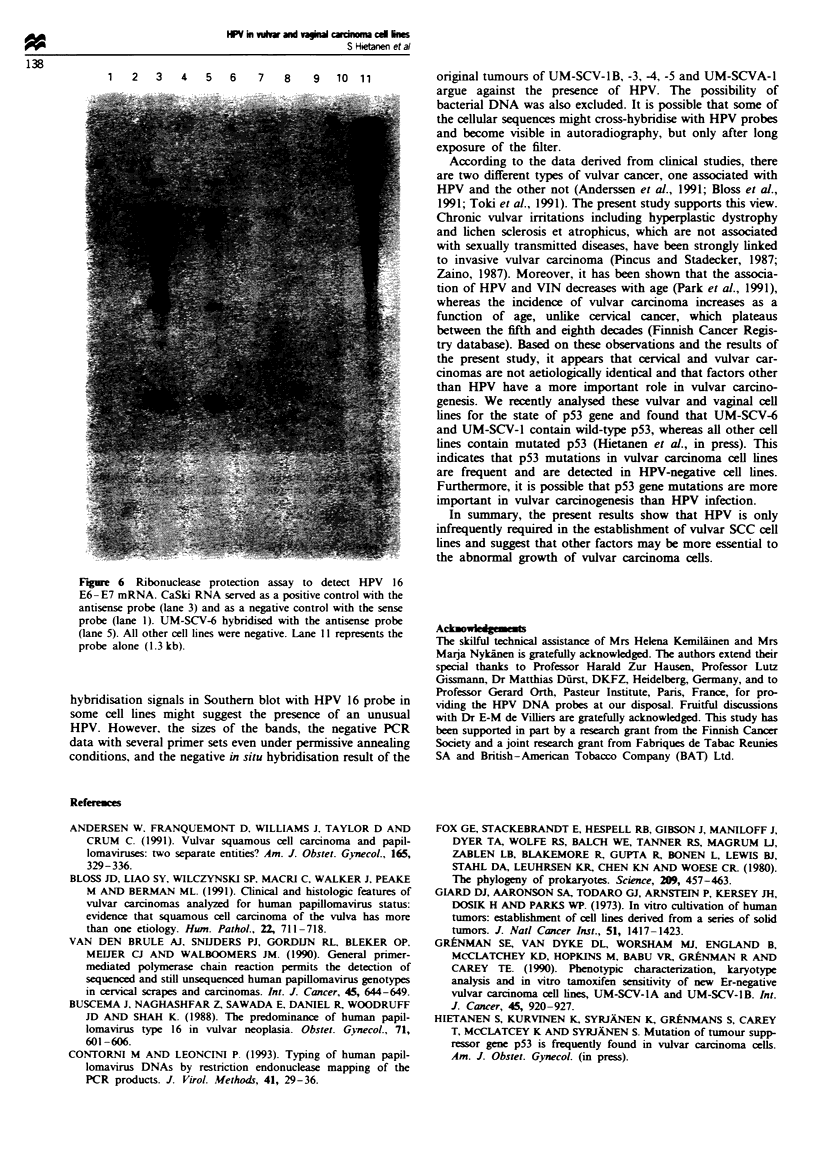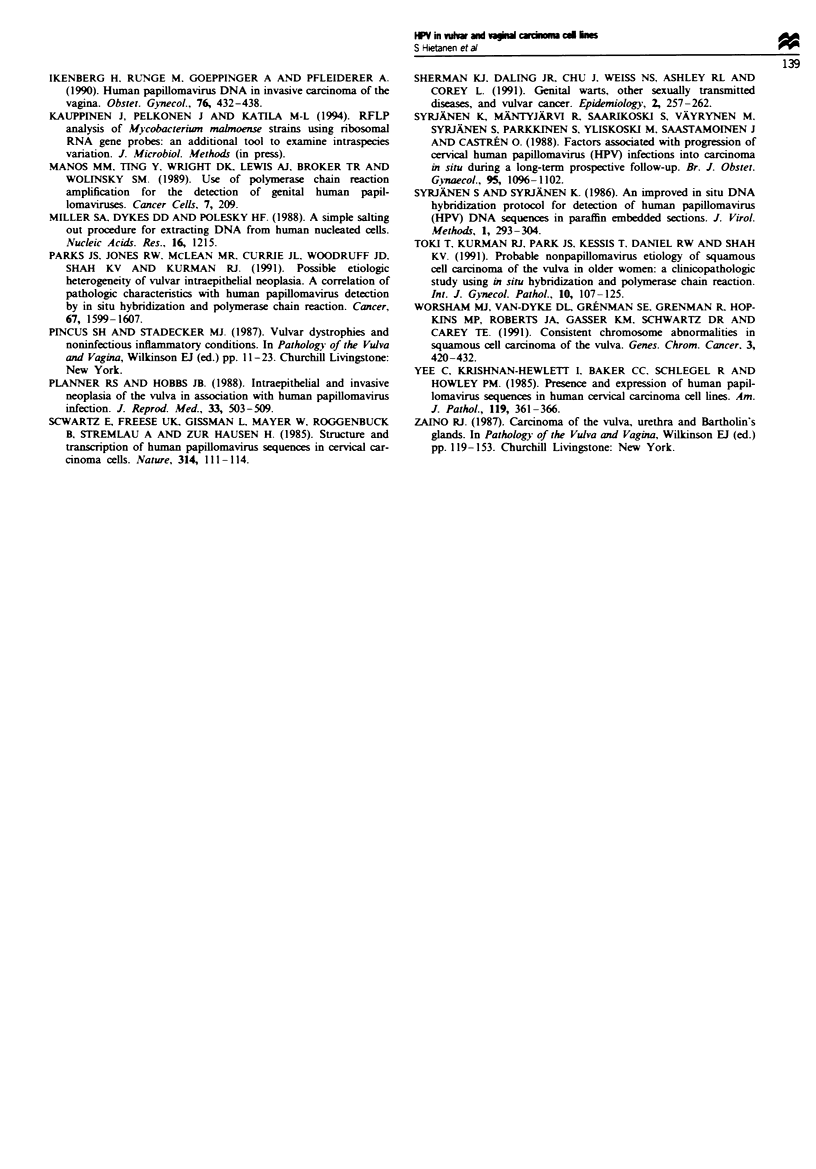# Human papillomavirus in vulvar and vaginal carcinoma cell lines.

**DOI:** 10.1038/bjc.1995.289

**Published:** 1995-07

**Authors:** S. Hietanen, S. Grénman, K. Syrjänen, K. Lappalainen, J. Kauppinen, T. Carey, S. Syrjänen

**Affiliations:** Department of Obstetrics and Gynecology, Turku University Central Hospital, Finland.

## Abstract

**Images:**


© 1995 Stockton Press All rights reserved 0007-0920/95 $12.00

### Human papillomavirus in vulvar and vaginal carcinoma cell lines

S Hietanen[1,2], S Grénman[1], K Syrjänen[3], K Lappalainen[2], J Kauppinen[4], T Carey[5] and S Syrjänen[2,6]

[1]*Department of Obstetrics and Gynecology, Turku University Central Hospital, Kiinamyllynkatu 2-4, 20520 Turku, Finland;* [2]*Medicity Research Laboratory, Turku University, Tykistökatu 6, 20520 Turku, Finland;* [3]*Department of Pathology, Kuopio University, PL 1627, 70211 Kuopio, Finland;* [4]*Department of Clinical Microbiology, Kuopio University, PL 1627, 70211 Kuopio, Finland;* [5]*Department of Otolaryngology/Head and Neck Surgery, Cancer Research Laboratory, University of Michigan, Ann Arbor, Michigan, USA;* [6]*Department of Oral Pathology, Institute of Dentistry, Turku University, Lemminkäisenkatu 2, 20520 Turku, Finland.*

**Summary** A number of reports associate human papillomavirus (HPV) with cervical cancer and cancer cell lines derived from this tumour type. Considerably fewer reports have focused on the role of HPV in carcinomas from other sites of female anogenital squamous epithelia. In this study we have tested for the presence of HPV in eight low-passage vulvar carcinoma cell lines and one extensively passaged cell line, A431. One cell line from a primary vaginal carcinoma was included. The presence of the HPV was evaluated by the polymerase chain reaction (PCR), by Southern blot analysis and by two-dimensional gel electrophoresis. General primer-mediated PCR was applied by using primers from the L1 region, E1 region and HPV 16 E7 region. Southern blot hybridisation was performed under low-stringency conditions ($T_m = -35°C$) using a whole genomic HPV 6/16/18 probe mixture and under high stringency conditions ($T_m = -18°C$) with the whole genomic probes of HPV 16 and 33. HPV 16 E6–E7 mRNA was assessed by ribonuclease protection assay (RPA). HPV was found in only one vulvar carcinoma cell line, UM-SCV-6. The identified type, HPV 16, was integrated in the cell genome and could be amplified with all primers used. Also E6–E7 transcripts were found in these cells. Five original tumour biopsies were available from the HPV-negative cell lines for *in situ* hybridisation. All these were HPV negative with both the HPV 6/16/18 screening probe mixture under low stringency and the HPV 16 probe under high stringency. The results indicate that vulvar carcinoma cell lines contain HPV less frequently than cervical carcinoma cell lines and suggest that a significant proportion of vulvar carcinomas may evolve by an HPV-independent mechanism.

**Keywords:** vulvar neoplasms; human papillomavirus; cancer cell line; carcinogenesis

Numerous studies have shown that most cervical carcinomas and cancer-derived cell lines contain human papillomavirus (HPV) genomes, usually integrated in the host cell genome. While HPV has been implicated in the aetiology of this particular type of cancer, data on the role of HPV at other genital sites are more fragmentary. There is some epidemiological evidence that HPV infection is a risk factor for vulvar carcinoma (Sherman *et al.*, 1991). HPV DNA has also been found in carcinoma samples of the vulva and vagina (Buscema *et al.*, 1988; Ikenberg *et al.*, 1990; Anderssen *et al.*, 1991; Bloss *et al.*, 1991; Toki *et al.*, 1991). HPV is known to infect the squamous epithelium of the vagina and vulva, and in doing so HPV may induce classical exophytic condylomata, flat lesions or low-grade intraepithelial neoplasia. Follow-up studies have shown that such lesions may progress to higher grades (Planner and Hobbs, 1988) in the same manner as in the cervix (Syrjänen *et al.*, 1988), although this occurs infrequently. Moreover, koilocytic atypia has been found within and in the vicinity of vulvar and vaginal intraepithelial neoplasia (VIN, VAIN) and in a subset of invasive neoplasia (Zaino *et al.*, 1987), which indicates some biological similarity with the pathogenesis of cervical neoplasia.

Many of the *in vitro* data evaluating the presence and physical state of HPV in squamous carcinoma of the female genital tract have been obtained using cancer cell lines derived from the uterine cervix. There are no previous studies on the presence of HPV DNA in cell lines derived from extracervical genital squamous carcinomas. The major reason for this has been the lack of suitable cell lines. We have recently established and characterised cell lines from human vulvar and vaginal carcinomas (Grénman *et al.*, 1990; Worsham *et al.*, 1991). These cell lines, as well as the long-established vulvar carcinoma cell line A431, have now been studied for the presence of HPV.

Correspondence: S Hietanen, Medicity Research Laboratory, Turku University, Tykistökatu 6, 20520 Turku, Finland
Received 23 September 1994; revised 19 January 1995; accepted 8 February 1995

### Materials and methods

### Cell lines

The establishment and characterisation of cell lines UM-SCV-1, 2, 3, 4, 5 and 6 have been described previously (Grénman *et al.*, 1990; Worsham *et al.*, 1991). Vulvar carcinoma cell line UM-SCV-7 and the vaginal carcinoma cell line UM-SCVA-1 were more recently established in our laboratory (S Grénman *et al.*, unpublished). The cell lines are summarised in Table I. Cultured cells were harvested with trypsin–EDTA. Total DNA was extracted from the cells by the method of Miller *et al.* (1988). Briefly, samples were lysed in 1 ml of 10 mM Tris (pH 8.3), 400 mM sodium chloride, 1% SDS, 2 mM EDTA and 0.3 mg $ml^{-1}$ proteinase K overnight at 37°C. Proteins were precipitated by adding 300 µl of saturated sodium chloride (approximately 6 M). After centrifugation, DNA was precipitated from the supernatant with ethanol. An HPV 16-positive cervical carcinoma cell line, CaSki, was used as a control.

### Southern blot hybridisation

Southern blotting followed the standard procedure. Briefly, 10 µg of restriction enzyme-digested or undigested cellular DNA was loaded into individual lanes and run into 1.0% agarose gels and transferred by Southern blotting to nylon filters (Gene Screen, Dupont, Boston, MA, USA) for subsequent hybridisation. Restriction endonucleases *Pst*I and *Ban*I, known to cut the HPV 16 genome, were used to digest the genomic DNA. Hybridisation was performed with vector-

**Table I** Cell lines and result of the HPV analysis

| Cell line | Origin | Age (years) | HPV in situ hybridisation of the original tumour | Passage of cell line | Detection of HPV in the cell line |
|---|---|---|---|---|---|
| UM-SCV-1A[a] | Vulva, primary tumour. SCC, grade II–III | 62 | NA | 14 | – |
| UM-SCV-1B[a] | Vulva, primary tumour, pleural effusion. SCC grade III | | – | 14 | – |
| UM-SCV-2[b] | Vulva, local recurrence. SCC, grade III | 86 | NA | 13 | – |
| UM-SCV-3[b] | Vulva, primary tumour. SCC, grade II | 66 | – | 19 | – |
| UM-SCV-4[b] | Vulva, primary tumour. SCC, grade? | 41 | – | 13 | – |
| UM-SCV-5[b] | Vulva, local recurrence. SCC, grade II | 60 | – | 9 | – |
| UM-SCV-6[b] | Vulva, primary tumour. SCC, grade? | 43 | NA | 13 | HPV 16 |
| UM-SCV-7[c] | Vulva, primary tumour. SCC, grade II–III | 77 | NA | 8 | – |
| A 431[d] | Vulva. SCC, grade? | 85 | NA | | – |
| UM-SCVA-1[c] | Vagina, local recurrence. SCC, grade II | 46 | – | 5 | – |

[a]Grénman *et al.* (1990). [b]Worsham *et al.* (1991). [c]Unpublished. [d]Giard *et al.* (1973). SCC, squamous cell carcinoma; NA, original tumour not available.

free, whole genomic HPV probes labelled with [α-$^{32}$P]dCTP by nick translation (Life Technologies, Gaithersburg, MD, USA). For screening, the samples were hybridised overnight under low-stringency conditions ($T_m = -35$°C), using a whole genomic HPV 6/16/18 probe mixture. After screening, the filters were rehybridised with HPV 16 and HPV 33 probes under high stringency ($T_m = -18$°C). The filters were exposed at −70°C for 1, 3 and 8 days. Before rehybridisation, the probe was removed from the filter by boiling with 0.1% SDS–1 mM EDTA for 2 min, followed by rapid cooling at 20°C. Removal of the hybridised probes was confirmed by exposing the filter for 24 h.

The absence of bacterial DNA in the cell lines was confirmed by hybridisation of the filters with 16S RNA gene probe. Bacterial ribosomal genes are highly conserved and universally presented among bacterial species, particularly the 16S rRNA (Fox *et al.*, 1980). the 16S rRNA gene probe was prepared by PCR using *Escherichia coli* DNA as a template to amplify a 1.3 kb product. The probe was labelled with $^{32}$P using a multiprime method. The primers used were TTTGAGCTCAGATTGAACGCTG and ATTGGATCCA-CGATTACTAGCG (Kauppinen *et al.*, 1994). *E. coli* DNA in decreasing concentrations (from 500 ng to 2 pg per lane), digested with *Hind*III, was used as a positive control.

### *Integration analysis*

The physical state of the HPV genome was studied with two-dimensional gel electrophoresis. A 5 μg sample of undigested genomic DNA was electrophoresed in a 0.4% agarose gel at 20 V for 17 h. The sample was incubated at 56°C before electrophoresis. The resulting lane was cut from the gel and recast in a 0.8% gel. This gel was electrophoresed at 90° to the original direction, using 70 V for 4 h. DNA was transferred to a filter and hybridised with $^{32}$P-labelled HPV 16 probe.

### *Polymerase chain reaction (PCR)*

The presence of HPV DNA was studied using PCR with four primer sets targeting the E1, L1 and HPV 16 E7 regions. The primers are shown in Table II. The reaction took place on 300 ng of genomic DNA in a 50 μl reaction volume. The PCR solution contained 5 μl of 10 × PCR buffer (50 mM potassium chloride, 10 mM Tris–HCl, pH 8.8, 1.5 mM magnesium chloride, 0.1% Triton X-100), 0.75 units of DynaZyme DNA polymerase (Finnzymes, Espoo, Finland), 200 μM of each deoxynucleotide triphosphate, 20 pmol of the primers and sterile water. The template DNA was first denatured for 4.5 min at 95°C and then exposed to 35 cycles of denaturation at 95°C for 30 s, annealing at 55°C for 50 s and extension at 72°C for 60 s in a thermal cycler (Cetus, Norwalk, CT, USA). The amplification was completed by a 4 min extension step at 72°C. We performed an additional PCR with GP5 and GP6 primers in more relaxed conditions: the template DNA was first denatured for 4.5 min at 95°C and then exposed to 40 cycles of denaturation at 94°C for 60 s, annealing at 40°C for 2 min and extension at 72°C for 90 s. HPV plasmid DNAs and DNA extracted from CaSki cells were used as positive controls. No DNA was added to the PCR solution of the negative controls. Both the undigested and digested PCR products were electrophoresed in 3% agarose gel (NuSieve, FMC BioProducts, Rockland, ME, USA). Bands were visualised by ethidium bromide staining. The PCR products with GP5–GP6 primers were transferred to a filter and hybridised using a mixture of whole genomic biotinylated HPV 6/16/18 probes under low-stringency conditions ($T_m = -35$°C). The hybrids were visualised with streptavidin–alkaline phosphatase complex using nitroblue tetrazolium as chromogen and 5-bromo-4-chloro-3-indolyl phosphate as substrate (Syrjänen and Syrjänen, 1986). β-Globin was amplified to ensure that the samples were appropriate for PCR. The sensitivity of the PCR was tested with MY09-11 primers and CaSki cells. We mixed 10 000, 1000, 100, 50, 10, 5 and 1 CaSki cells with 300 ng of DNA extracted from normal human fibroblasts and performed the PCR in the more stringent conditions as described above. The PCR was still positive with one CaSki cell (data not shown).

### *Ribonuclease protection assay (RPA)*

Samples showing any bands in Southern blot hybridisation were analysed for the presence of HPV 16 E6–E7 RNA transcripts. The analysis included cell lines UM-SCV-1A, -1B, -4, -5, -6, A431 and UM-SCVA-1. After DNAse (RNAse-free) (Promega, Madison, WI, USA) treatment, 10 μg of total cellular RNA was analysed with the RPAII kit (Ambion, TX, USA) according to the manufacturer's instructions. The RNA samples were hybridised with the RNA probe spanning from the upstream regulatory region to the entire E6–E7 open reading frames (ORFs) of HPV 16 (nucleotide positions 7454–880). The $^{32}$P-labelled RNA probes were generated in the Bluescript transcription vector using Riboprobe Gemini Systems (Promega), with T3 or T7 RNA polymerase according to the standard protocol supplied by the manufacturer. The specific activity of the RNA probe was 8 × 10$^{8}$ c.p.m. μg$^{-1}$. The sense orientation of the

**Table II** Sequences of the oligonucleotide primers used for amplification of HPV and β-Globin

| *Target* | *Primer* | *Sequence* | *Size (bp)* |
|---|---|---|---|
| HPV L1 | MY 09[a] | 5'-CGTCCMARRGGAWACTGATC-3' | 448–454 |
| | MY11 | 5'-GCMCAGGGWCATAAYAATGG-3' | |
| HPV L1 | GP5[b] | 5'-TTTGTTACTGTGGTAGATAC-3' | 140–150 |
| | GP6 | 5'-GAAAAATAAACTGTAAATCS-3' | |
| HPV E1 | p1E1[c] | 5'-TATGGCTATTCTGAAGTGGAA-3' | 526–583 |
| | p2E1 | 5'-TTGATATACCTGTTCTAAACCA-3' | |
| HPV 16 E7 | E7A1 | 5'-GGATCCTACATTGCATGAATATATG-3' | 272 |
| | E7A2 | 5'-CTGCAGATGGGGCACACAATTCCTA-3' | |
| β-Globin | β-Globin 1 | 5'-ACACAACTGTGTTCACTAGC-3' | 110 |
| | β-Globin 2 | 5'-CAACTTCATCCACGTTCACC-3' | |

M = A + C, R = A + T, W = A + T, Y = C + T. [a]Manos *et al.* (1989). [b]van den Brule *et al.* (1990). [c]Contorni and Leoncini (1993).

probe served as a negative control. The hybridisation was performed overnight with $^{32}$P-labelled probe (specific activity $2.5 \times 10^5$ c.p.m). After RNAse digestion and ethanol precipitation the samples were analysed in 5% polyacrylamide gel in 8 M urea. The gel was exposed overnight at −70°C.

### In situ *hybridisation of the original tumour samples*

Original tumour samples of cell lines UM-SCV-1B, -3, -4, -5 and UM-SCVA-1 were available for HPV *in situ* hybridisation. The hybridisation was performed with the HPV 6/16/18 mixture and HPV 16 probe under low and high stringency respectively (Syrjänen and Syrjänen, 1986).

### Results

### *Southern blot analysis and two-dimensional gel electrophoresis*

Only UM-SCV-6 hybridised clearly after 1 day's exposure to the HPV 6/16/18 probe mixture under low stringency. Five other vulvar cell lines (UM-SCV-1A, -1B, -4, -5 and A431) and the vaginal cell line (UM-SCVA-1) showed some bands after 8 days' exposure (Figures 1 and 2). The restriction pattern of UM-SCV-6 was similar to that found with DNA extracted from CaSki cells, indicating the presence of HPV type 16. This was confirmed by rehybridisation with HPV 16 probe under high stringency (Figure 3). The other vulvar cell lines and the vaginal cell line with positive bands under low-stringency conditions also showed some faint bands after hybridisation with HPV 16 probe under high stringency conditions after long exposure (Figures 3 and 4). However, the band sizes were less than expected for HPV and the restriction pattern did not fit any of the known HPV types. Also, the original tumour biopsies from these cell lines were HPV DNA negative by *in situ* hybridisation (Table I). None of the cell lines hybridised with the HPV 33 probe under high-stringency conditions (data not shown). The copy number of the HPV genome in UM-SCV-6 cells was some 200–300 as judged from the comparison of hybridisation signals to the CaSki cells (500–600 HPV 16 copies). HPV 16 DNA in UM-SCV-6 cell line was integrated, since undigested samples showed high molecular weight signals (Figure 3). The integration was confirmed by two-dimensional gel electrophoresis. Intense hybridisation signals were detected with slowly migrating high molecular weight DNA, which are compatible with integrated DNA sequences. No circular sequences were detectable, indicating that UM-SCV-6 contained no episomal sequences (Figure 5). The absence of bacterial sequences in DNAs extracted from the cell lines was confirmed by hybridising the filters with bacterial 16S rRNA gene probe. *E. coli* DNA on the positive control filter yielded detectable signals down to the concentration of 200 pg. All cell lines were negative.

**Figure 1** Autoradiogram of a Southern blot of cell lines UM-SCV-4, -5, -6 and A431. Cellular DNA (10 μg) was electrophoretically separated, blotted and hybridised with an HPV 6/16/18 probe mixture under low stringency. Exposure of the filter was 8 days. In each case, lane A contained *Pst*I-digested DNA, lane B *Ban*I-digested DNA and lane C undigested DNA. Molecular weight markers are *Hind*III fragments of phage lambda DNA.

### *Polymerase chain reaction*

β-Globin was amplified in all cell lines. PCR with all three primer sets from L1 and E1 ORF amplified only DNA from UM-SCV-6 cells. All other cell lines were negative. As the Southern blot showed positive hybridisation signals with HPV 16 under high stringency not only with UM-SCV-6, but also with UM-SCV-1A, -1B, -4, -5, A431 and UM-SCVA-1, these cell lines were reanalysed with PCR using HPV 16 E7 primers. Only UM-SCV-6 yielded positive amplification. In order to detect other HPVs which might be less homologous with the GP5 and GP6 primers, we performed an additional PCR under more relaxed conditions. UM-SCV-6 and CaSki were positive, but all others remained negative even after hybridisation of the PCR products with a probe mixture of HPV 6/16/18 under low stringency (data not shown).

### *HPV 16 E7-E6 mRNA analysis*

HPV 16 E7-E6 transcripts were found only in UM-SCV-6 and CaSki cells as determined by ribonuclease protection assay. All other cell lines were negative. Hybridisation with the sense probe was negative with both CaSki and UM-SCV-5 cells (Figure 6).

### Discussion

Most cervical cancer biopsies and cell lines derived from cervical cancer contain integrated HPV and have shown transcripts from E6 and E7 ORFs of the persisting HPV DNA (Schwartz et al., 1985; Yee et al., 1985). The role of HPV in the carcinogenesis of other sites of female genital squamous epithelium has been less clearly defined. In this study we analysed the presence of HPV in one vaginal and nine vulvar carcinoma cell lines. Only one vulvar cell line, UM-SCV-6, contained HPV. This cell line contains integrated HPV 16 and E6-E7 ORFs are transcribed. The faint

**Figure 2** Cell lines UM-SCV-7 (lane 1), UM-SCVA-1 (lane 2), UM-SCV-1A (lane 3), UM-SCV-1B (lane 4), UM-SCV-2 (lane 5) and UM-SCV-3 (lane 6) hybridised under low stringency with a mixture of probes HPV 6, 16 and 18. DNA was cut with *Pst*I. Exposure: 8 days.

**Figure 3** Same filter as in Figure 1 hybridised with HPV 16 under high stringency. Exposure: 8 days.

**Figure 4** Same filter as in Figure 3 now hybridised with HPV 16 probe under high stringency. Exposure: 8 days.

**Figure 5** Two-dimensional gel electrophoresis of the UM-SCV-6 cell line. HPV 16 DNA is used as a hybridisation probe under high stringency. The probe hybridises with linearised DNA which migrates slowly with high molecular weight DNA, indicating that this cell line contains only integrated HPV 16 DNA.

**Figure 6** Ribonuclease protection assay to detect HPV 16 E6-E7 mRNA. CaSki RNA served as a positive control with the antisense probe (lane 3) and as a negative control with the sense probe (lane 1). UM-SCV-6 hybridised with the antisense probe (lane 5). All other cell lines were negative. Lane 11 represents the probe alone (1.3 kb).

hybridisation signals in Southern blot with HPV 16 probe in some cell lines might suggest the presence of an unusual HPV. However, the sizes of the bands, the negative PCR data with several primer sets even under permissive annealing conditions, and the negative *in situ* hybridisation result of the original tumours of UM-SCV-1B, -3, -4, -5 and UM-SCVA-1 argue against the presence of HPV. The possibility of bacterial DNA was also excluded. It is possible that some of the cellular sequences might cross-hybridise with HPV probes and become visible in autoradiography, but only after long exposure of the filter.

According to the data derived from clinical studies, there are two different types of vulvar cancer, one associated with HPV and the other not (Anderssen *et al.*, 1991; Bloss *et al.*, 1991; Toki *et al.*, 1991). The present study supports this view. Chronic vulvar irritations including hyperplastic dystrophy and lichen sclerosis et atrophicus, which are not associated with sexually transmitted diseases, have been strongly linked to invasive vulvar carcinoma (Pincus and Stadecker, 1987; Zaino, 1987). Moreover, it has been shown that the association of HPV and VIN decreases with age (Park *et al.*, 1991), whereas the incidence of vulvar carcinoma increases as a function of age, unlike cervical cancer, which plateaus between the fifth and eighth decades (Finnish Cancer Registry database). Based on these observations and the results of the present study, it appears that cervical and vulvar carcinomas are not aetiologically identical and that factors other than HPV have a more important role in vulvar carcinogenesis. We recently analysed these vulvar and vaginal cell lines for the state of p53 gene and found that UM-SCV-6 and UM-SCV-1 contain wild-type p53, whereas all other cell lines contain mutated p53 (Hietanen *et al.*, in press). This indicates that p53 mutations in vulvar carcinoma cell lines are frequent and are detected in HPV-negative cell lines. Furthermore, it is possible that p53 gene mutations are more important in vulvar carcinogenesis than HPV infection.

In summary, the present results show that HPV is only infrequently required in the establishment of vulvar SCC cell lines and suggest that other factors may be more essential to the abnormal growth of vulvar carcinoma cells.

**Acknowledgements**

The skilful technical assistance of Mrs Helena Kemiläinen and Mrs Marja Nykänen is gratefully acknowledged. The authors extend their special thanks to Professor Harald Zur Hausen, Professor Lutz Gissmann, Dr Matthias Dürst, DKFZ, Heidelberg, Germany, and to Professor Gerard Orth, Pasteur Institute, Paris, France, for providing the HPV DNA probes at our disposal. Fruitful discussions with Dr E-M de Villiers are gratefully acknowledged. This study has been supported in part by a research grant from the Finnish Cancer Society and a joint research grant from Fabriques de Tabac Reunies SA and British-American Tobacco Company (BAT) Ltd.

**References**

ANDERSEN W, FRANQUEMONT D, WILLIAMS J, TAYLOR D AND CRUM C. (1991). Vulvar squamous cell carcinoma and papillomaviruses: two separate entities? *Am. J. Obstet. Gynecol.*, **165,** 329-336.

BLOSS JD, LIAO SY, WILCZYNSKI SP, MACRI C, WALKER J, PEAKE M AND BERMAN ML. (1991). Clinical and histologic features of vulvar carcinomas analyzed for human papillomavirus status: evidence that squamous cell carcinoma of the vulva has more than one etiology. *Hum. Pathol.*, **22,** 711-718.

VAN DEN BRULE AJ, SNIJDERS PJ, GORDIJN RL, BLEKER OP, MEIJER CJ AND WALBOOMERS JM. (1990). General primer-mediated polymerase chain reaction permits the detection of sequenced and still unsequenced human papillomavirus genotypes in cervical scrapes and carcinomas. *Int. J. Cancer*, **45,** 644-649.

BUSCEMA J, NAGHASHFAR Z, SAWADA E, DANIEL R, WOODRUFF JD AND SHAH K. (1988). The predominance of human papillomavirus type 16 in vulvar neoplasia. *Obstet. Gynecol.*, **71,** 601-606.

CONTORNI M AND LEONCINI P. (1993). Typing of human papillomavirus DNAs by restriction endonuclease mapping of the PCR products. *J. Virol. Methods*, **41,** 29-36.

FOX GE, STACKEBRANDT E, HESPELL RB, GIBSON J, MANILOFF J, DYER TA, WOLFE RS, BALCH WE, TANNER RS, MAGRUM LJ, ZABLEN LB, BLAKEMORE R, GUPTA R, BONEN L, LEWIS BJ, STAHL DA, LEUHRSEN KR, CHEN KN AND WOESE CR. (1980). The phylogeny of prokaryotes. *Science*, **209,** 457-463.

GIARD DJ, AARONSON SA, TODARO GJ, ARNSTEIN P, KERSEY JH, DOSIK H AND PARKS WP. (1973). In vitro cultivation of human tumors: establishment of cell lines derived from a series of solid tumors. *J. Natl Cancer Inst.*, **51,** 1417-1423.

GRÉNMAN SE, VAN DYKE DL, WORSHAM MJ, ENGLAND B, MCCLATCHEY KD, HOPKINS M, BABU VR, GRÉNMAN R AND CAREY TE. (1990). Phenotypic characterization, karyotype analysis and in vitro tamoxifen sensitivity of new Er-negative vulvar carcinoma cell lines, UM-SCV-1A and UM-SCV-1B. *Int. J. Cancer*, **45,** 920-927.

HIETANEN S, KURVINEN K, SYRJÄNEN K, GRÉNMANS S, CAREY T, MCCLATCEY K AND SYRJÄNEN S. Mutation of tumour suppressor gene p53 is frequently found in vulvar carcinoma cells. *Am. J. Obstet. Gynecol.* (in press).

IKENBERG H, RUNGE M, GOEPPINGER A AND PFLEIDERER A. (1990). Human papillomavirus DNA in invasive carcinoma of the vagina. *Obstet. Gynecol.*, **76**, 432–438.

KAUPPINEN J, PELKONEN J AND KATILA M-L (1994). RFLP analysis of *Mycobacterium malmoense* strains using ribosomal RNA gene probes: an additional tool to examine intraspecies variation. *J. Microbiol. Methods* (in press).

MANOS MM, TING Y, WRIGHT DK, LEWIS AJ, BROKER TR AND WOLINSKY SM. (1989). Use of polymerase chain reaction amplification for the detection of genital human papillomaviruses. *Cancer Cells*, **7**, 209.

MILLER SA, DYKES DD AND POLESKY HF. (1988). A simple salting out procedure for extracting DNA from human nucleated cells. *Nucleic Acids. Res.*, **16**, 1215.

PARKS JS, JONES RW, MCLEAN MR, CURRIE JL, WOODRUFF JD, SHAH KV AND KURMAN RJ. (1991). Possible etiologic heterogeneity of vulvar intraepithelial neoplasia. A correlation of pathologic characteristics with human papillomavirus detection by in situ hybridization and polymerase chain reaction. *Cancer*, **67**, 1599–1607.

PINCUS SH AND STADECKER MJ. (1987). Vulvar dystrophies and noninfectious inflammatory conditions. In *Pathology of the Vulva and Vagina*, Wilkinson EJ (ed.) pp. 11–23. Churchill Livingstone: New York.

PLANNER RS AND HOBBS JB. (1988). Intraepithelial and invasive neoplasia of the vulva in association with human papillomavirus infection. *J. Reprod. Med.*, **33**, 503–509.

SCWARTZ E, FREESE UK, GISSMAN L, MAYER W, ROGGENBUCK B, STREMLAU A AND ZUR HAUSEN H. (1985). Structure and transcription of human papillomavirus sequences in cervical carcinoma cells. *Nature*, **314**, 111–114.

SHERMAN KJ, DALING JR, CHU J, WEISS NS, ASHLEY RL AND COREY L. (1991). Genital warts, other sexually transmitted diseases, and vulvar cancer. *Epidemiology*, **2**, 257–262.

SYRJÄNEN K, MÄNTYJÄRVI R, SAARIKOSKI S, VÄYRYNEN M, SYRJÄNEN S, PARKKINEN S, YLISKOSKI M, SAASTAMOINEN J AND CASTRÉN O. (1988). Factors associated with progression of cervical human papillomavirus (HPV) infections into carcinoma *in situ* during a long-term prospective follow-up. *Br. J. Obstet. Gynaecol.*, **95**, 1096–1102.

SYRJÄNEN S AND SYRJÄNEN K. (1986). An improved in situ DNA hybridization protocol for detection of human papillomavirus (HPV) DNA sequences in paraffin embedded sections. *J. Virol. Methods*, **1**, 293–304.

TOKI T, KURMAN RJ, PARK JS, KESSIS T, DANIEL RW AND SHAH KV. (1991). Probable nonpapillomavirus etiology of squamous cell carcinoma of the vulva in older women: a clinicopathologic study using *in situ* hybridization and polymerase chain reaction. *Int. J. Gynecol. Pathol.*, **10**, 107–125.

WORSHAM MJ, VAN-DYKE DL, GRÉNMAN SE, GRENMAN R, HOPKINS MP, ROBERTS JA, GASSER KM, SCHWARTZ DR AND CAREY TE. (1991). Consistent chromosome abnormalities in squamous cell carcinoma of the vulva. *Genes. Chrom. Cancer*, **3**, 420–432.

YEE C, KRISHNAN-HEWLETT I, BAKER CC, SCHLEGEL R AND HOWLEY PM. (1985). Presence and expression of human papillomavirus sequences in human cervical carcinoma cell lines. *Am. J. Pathol.*, **119**, 361–366.

ZAINO RJ. (1987). Carcinoma of the vulva, urethra and Bartholin's glands. In *Pathology of the Vulva and Vagina*, Wilkinson EJ (ed.) pp. 119–153. Churchill Livingstone: New York.