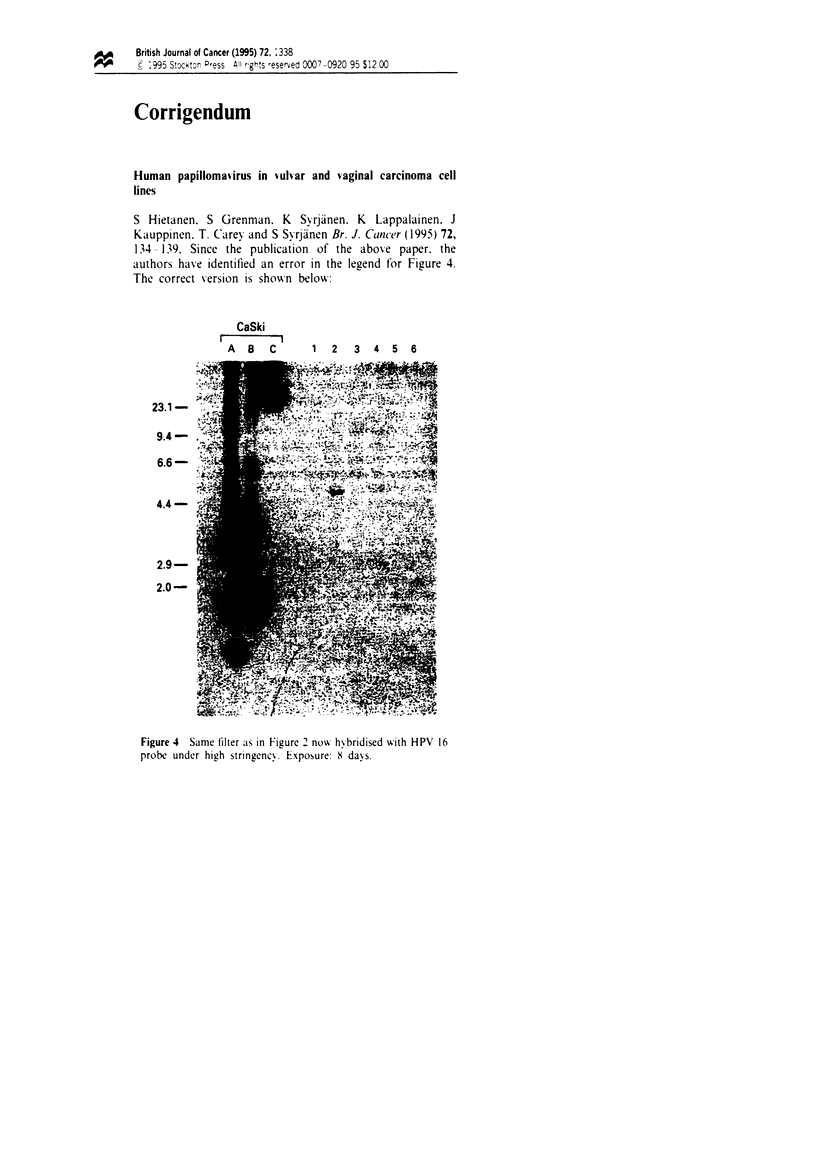# Human papillomavirus in vulvar and vaginal carcinoma cell lines

**Published:** 1995-11

**Authors:** 

## Abstract

**Images:**


British Journal of Cancer (1995) 72, 1338

© 1995 Stockton Press All rights reserved 0007-0920/95 $12.00

### Corrigendum

### Human papillomavirus in vulvar and vaginal carcinoma cell lines

S Hietanen, S Grenman, K Syrjänen, K Lappalainen, J Kauppinen, T. Carey and S Syrjänen *Br. J. Cancer* (1995) **72**, 134–139. Since the publication of the above paper, the authors have identified an error in the legend for Figure 4. The correct version is shown below:

**Figure 4** Same filter as in Figure 2 now hybridised with HPV 16 probe under high stringency. Exposure: 8 days.